# 4049 Evaluating Miami CTSI’s Pilot, Translational, and Clinical Studies Program using research success measures and CTSA Common Metrics

**DOI:** 10.1017/cts.2020.234

**Published:** 2020-07-29

**Authors:** Rosalina Das, Patricia Avissar, Jessica Diaz, Sheela C Dominguez, Barry S. Issenberg, Dalton W. Dietrich, Ralph L. Sacco

**Affiliations:** 1 University of Miami Clinical and Translational Science Institute; 2 University of Miami

## Abstract

OBJECTIVES/GOALS: The goal of this project was to a) evaluate the first five years of Miami CTSI’s Pilot Translational and Clinical Studies Program using outcome measures that quantify research productivity augmented by the CTSA Common Metrics; and b) use the results to shape future program management. METHODS/STUDY POPULATION: Pilot Program applicant and awardee demographic data were collected during the first 5-year cycle of the Miami CTSI grant. Projects were categorized into the translation spectrum based on type of research using published guidelines. Research productivity from funded pilot projects were tracked annually using internal institutional grant award databases and external databases such as PubMed and NIH Reporter. CTSA Common Metrics were tracked using the Results Based Accountability framework. Relative Citation Ratio (RCR), NIH percentile and translation impact of pilot project publications were determined using the iCite tool (NIH Office of Portfolio Analysis). RESULTS/ANTICIPATED RESULTS: The Miami CTSI’s Pilot Award Program demonstrated notable success in its first five years. Of the twenty-two projects that were funded during that time period, 45% led to follow-on funding for a total of $17.2M—a strong return on investment of 15:1. Further, 77% of awardees had at least one publication. A total of four patents and 43 publications resulted directly from the funded projects. The mean RCR for all publications was 2.7, weighted RCR was 99.87, and nine papers were been cited by clinical documents. Overall, 63% of the projects were classified as T1/T2 (pre-clinical/clinical research) and 37% as T3/T4 (post-clinical translational research/public health). DISCUSSION/SIGNIFICANCE OF IMPACT: Miami CTSI’s Pilot Award Program demonstrated success in scholarly output, follow on funding, and scientific impact. These results will serve as benchmarks going forward and will allow the CTSI to leverage program strengths in collaborating with other institutional internal award mechanisms.

